# How does bark contribution to postural control change during tree ontogeny? A study of six Amazonian tree species

**DOI:** 10.1093/jxb/eraa070

**Published:** 2020-02-13

**Authors:** Romain Lehnebach, Tancrède Alméras, Bruno Clair

**Affiliations:** 1 LMGC, Université de Montpellier, CNRS, Montpellier, France; 2 UGent-Woodlab, Laboratory of Wood Technology, Department of Environment, Gent University, Gent, Belgium; 3 CNRS, UMR Ecologie des Forêts de Guyane (EcoFoG), AgroParisTech, CIRAD, INRA, Université des Antilles, Université de Guyane, Campus agronomique, Kourou cedex, France; 4 INRAE-University Clermont Auvergne, France

**Keywords:** Allometry, bark density, bark residual strain, bark thickness, inner bark, mechanical stress, ontogeny, sclereids, tropical trees

## Abstract

Recent works revealed that bark is able to produce mechanical stress to control the orientation of young tilted stems. Here we report how the potential performance of this function changes with stem size in six Amazonian species with contrasted bark anatomy. The potential performance of the mechanism depends both on the magnitude of bark stress and the relative thickness of the bark. We measured bark longitudinal residual strain and density, and the allometric relationship between bark thickness and stem radius over a gradient of tree sizes. Constant tensile stress was found in species that rely on bark for the control of stem orientation in young stages. Other species had increasing compressive stress, associated with increasing density attributed to the development of sclereids. Compressive stress was also associated with low relative bark thickness. The relative thickness of bark decreased with size in all species, suggesting that a reorientation mechanism based on bark progressively performs less well as the tree grows. However, greater relative thickness was observed in species with more tensile stress, thereby evidencing that this reduction in performance is mitigated in species that rely on bark for reorientation.

## Teaser

During ontogeny, species that rely on bark for posture control maintain thick bark and constant tensile stress, while other species have relatively thinner bark and increasing compressive stress.

## Introduction

Bark is a multifunctional tissue (Angyalossy *et al.*, 2016; Rosell, 2019). Its functions include transport of photosynthates (Evert, 2006), storage of carbohydrates and water (Rosell and Olson, 2014), defense against physical and biological aggression (Pausas, 2015), and mechanical support (Niklas, 1999; Rosell and Olson, 2014). The mechanical function was long thought to be limited to contributing to stem stiffness. Stiffness is a purely ‘passive’ mechanical property; it quantifies the ability of the stem to resist movement caused by external forces, as opposed to ‘active’ mechanical functions, namely the ability to generate internal forces that cause stem movement. Recent works reported the involvement of inner bark (i.e. secondary phloem), hereafter simply bark, in the active control of stem orientation (Clair *et al.*, 2019). Bark fulfills this function by generating mechanical stress during stem secondary growth. Combined with a source of asymmetry (here eccentric growth), this active generation of mechanical stress results in a bending moment capable of altering stem curvature and orientation (Clair *et al.*, 2019).

Measuring and interpreting the state of stress of bark are, however, not easy. In the above-mentioned work (Clair *et al.*, 2019), the mechanical function of bark was demonstrated by growing young stems in a tilted position. At the end of the experiment, the change in stem curvature that occurred when the stem is released from the stake was measured and compared with the change in stem curvature that occurred when the bark was removed from the stem. These measurements of curvature informed us about the imbalance in stress in the bark ring, namely the difference in stress between the upper and lower sides of the tilted stem.

This method cannot easily be applied to adult trees, but ‘direct’ measurement of stress is possible by measuring released strains (Clair *et al.*, 2019; Lehnebach *et al.*, 2019). The measurement consists of recording the strain that occurs in bark when it is mechanically isolated from surrounding tissues, a method routinely used to assess the mechanical state of the most recent wood layers (Fournier *et al.*, 1994; Ruelle *et al.*, 2006; Clair *et al.*, 2013). Interpreting this measurement is more complex than for wood. Indeed, wood located at the tree periphery was formed recently and thus has a short mechanical history. Therefore, its state of stress mainly results from its own maturation process. The state of stress of bark is more complex to interpret because bark is made of successive layers that have been produced throughout the life of the tree, involving a long and possibly complicated mechanical history. This is particularly the case for tilted stems, in which the mechanical state of the bark is modified not only by stem secondary growth, but also by the changes in curvature that occurred in response to the effect of self-weight and the action of wood (see note S1 in Clair *et al.*, 2019).

Measuring the residual strain in vertical trees avoids these complications and enables evaluation of the average stress of bark. We previously performed measurements on vertical trees belonging to 15 tree species (Lehnebach *et al.*, 2019) and our results revealed contrasted states of stress among species, ranging from strong tension to strong compression. These states of stress were interpreted in the light of bark anatomical properties (i.e. cell types and the organization of the tissues) and processes of stem secondary growth. The state of tension is typically associated with the presence of phloem fibers organized like a trellis (Lehnebach *et al.*, 2019). When wood grows, the perimeter of bark is forced to increase (Whitmore, 1962; Evert, 2006), resulting in a state of tension in the tangential direction (Clair *et al.*, 2019). Because of the particular trellis structure, the tangential tension is redirected in the longitudinal direction, resulting in longitudinal tension (a more detailed explanation is given in Clair *et al.*, 2019). The state of compression does not depend on wood radial growth but is typically associated with a large quantity of sclereids in the bark. Sclereids have thick cell walls, and are typically associated with denser bark (Carmo *et al.*, 2016; Lehnebach *et al.*, 2019). The quantity of sclereids in bark is negatively correlated with susceptibility to beetles, and is generally acknowledged to be a deterrent against herbivores (Wainhouse *et al.*, 1990; Thorpe and Day, 2002). Sclereids derive from the differentiation of parenchyma cells (Prislan *et al.*, 2012). During their development, these cells swell in all directions, resulting in compressive stress in the longitudinal direction.

An important issue is the capacity of bark to maintain its biomechanical function as the tree grows. Indeed, direct evidence that bark is capable of bending a tree stem has only been provided in greenhouse experiments, using small trees (Clair *et al.*, 2019). Here we address the question of how the state of stress of bark changes during stem ontogeny. The performance (which can be measured as the change in curvature occurring in a tilted plant) directly depends on two factors: the magnitude of stress in the bark and its geometrical contribution. In this way, we assessed how the state of stress of bark changes with ontogeny. As the geometrical contribution of bark generally decreases during ontogeny (Niklas, 1999; Rosell and Olson, 2014), the biomechanical performance of this mechanism is challenged when the tree grows. We therefore also aimed to assess the allometry between bark thickness and stem radius, and to investigate the link between this allometry and the state of stress. As bark is an indicator of bark stress (Lehnebach *et al.*, 2019), we also assessed variations in bark density as a function of stem size, and link the changes in both density and residual strain changes that occur during growth. The rationale of each experiment and the underlying questions are described in Table 1.

**Table 1.** The rationale of the study

Question	Methodology and analysis	Expected results	Figures
How does the state of stress of bark change with ontogeny?	Regression between bark residual strain and stem diameter.	Tensile stress increases in species relying on bark for postural control.	Fig. 5; Supplementary Table S2
Do species that differ in their bark mechanical behavior present different bark allometries?	Allometric analysis (standard major axis regression) between bark thickness and stem radius and wood thickness.	Species with tensile stress produce more bark (i.e. higher allometric exponent) than species with compressive stress.	Figs 2, 3; Supplementary Fig. S1; Table 4
Does bark density vary with ontogeny? Are bark density changes related to changes in the state of stress of bark?	Regression between bark density and stem size.	As both high bark density and compressive stress are associated with high level of bark sclerification (Lehnebach, 2019), tensile stress is associated with low bark density.	Fig. 4

Each question is described with the associated methodology, analysis, and expected results. The table also refers the reader to the figures and tables that give the the actual results associated with each question.

## Materials and methods

### Plant material and sampling

**Fig. 1.**
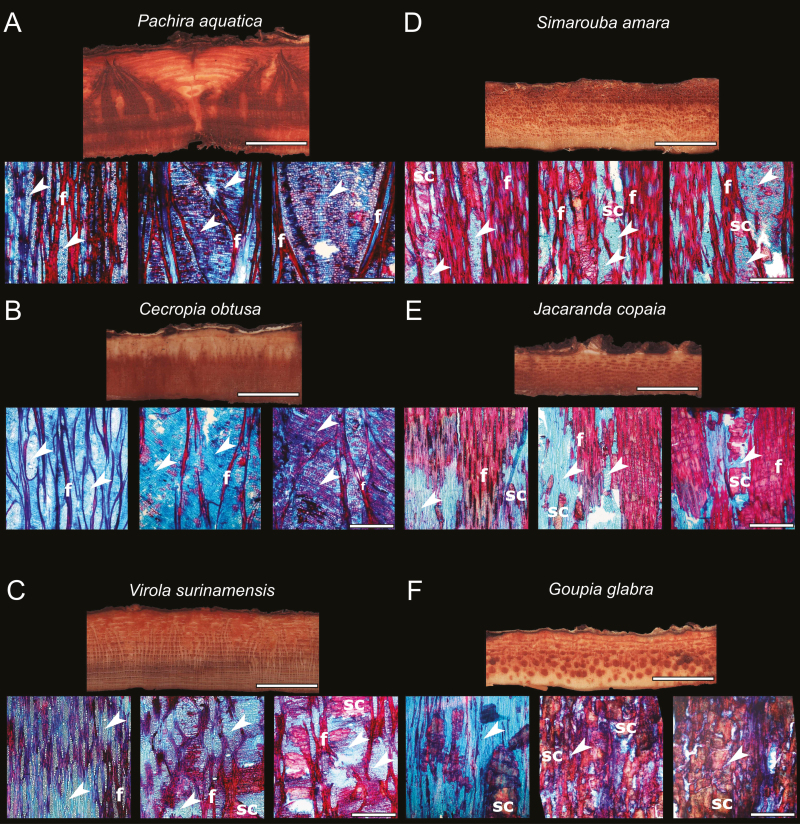
Macro- and microanatomical characteristics of the bark in six tropical tree species in the tropical rainforest of French Guiana. For each species, the macrostructural features of the bark in cross-section are presented (panel top), with the cambium at the bottom and the outer bark at the top (scale bar=1 cm) as well as three longitudinal microsections (panel bottom) of the inner bark at increasing distance from the cambium (from left to right) (scale bar=1 mm). In *Pachira*, *Cecropia*, *Virola*, and *Simarouba* (A–D), the bark presents fibers (f) organized like a trellis and enlarging rays (white arrowhead) from the cambium outward. The bark of *Jacaranda* (E) presents fibers (f) that are not organized like a trellis and sclereids (sc). The bark of *Goupia* (F) shows no fibers but a substantial amount of sclereids. Sclereids were observable whatever the position in the bark in *Simarouba*, *Jacaranda*, and *Goupia* (E, F) while they were observed in the outer part of the bark in *Virola* (C) and were absent in *Pachira* and *Cecropia* (A and B). Micrographs of *Pachira* and *Cecropia* (A and B) and *Virola*, *Simarouba*, *Jacaranda*, and *Goupia* (C, D–F) were originally published by Clair *et al.* (2019) and Lehnebach *et al.* (2019), respectively. (This figure is available in color at *JXB* online.)

Based on previous knowledge (Clair *et al.*, 2019; Lehnebach *et al.*, 2019), we selected six widespread Amazonian tree species that have contrasted mechanical states of bark at the adult stage as well as contrasted anatomical structure (Fig. 1). Among the six species, three species (*Pachira aquatica*, *Cecropia obtusa*, and *Virola surinamensis*) have bark with a peculiar trellis fiber network and associated tensile stress (Fig. 1A–C). One species (*Simarouba amara*) presents a less marked trellis and light tensile stress (Fig. 1D). The other two species have bark with compressive stress and either fibers that are not organized like a trellis (*Jacaranda copaia*, Fig. 1E) or no fibers (*Goupia glabra*, Fig. 1F)

As studying allometry requires log-transformed variables, our range of sampling sizes was divided into five classes corresponding to an increasing range of stem diameters (0–2.5, 2.5–5, 5–10, 10–20, and 20–40 cm). We sampled four trees per class and per species—a total of 20 trees per species. Five species out of six were sampled along the edge of the secondary forest between the cities of Kourou and Sinnamary in French Guiana, and the riparian species *P. aquatica* was sampled along the banks of the Kourou River.

### Measurement of inner bark residual longitudinal strain

Bark residual longitudinal strain (BRLS) was measured using the same methodology as in Lehnebach *et al.* (2019). After removing the outer bark from an area measuring 3×3 cm, an HBM DDL-1 extensometer sensor, consisting of two pins, was inserted in the secondary phloem along the longitudinal direction of the stem. The sensor was connected to a strain indicator (Vishay P3) to record the strain. The longitudinal strain of the bark was released by sawing two tangential grooves (one above and one below the sensor) at a distance of 4–7 mm from the corresponding pin. The BRLS was defined as the value recorded after making the second groove. The value is negative if the stress is tensile and positive if the stress is compressive. As trees are never perfectly vertical, the stress in bark is affected by bending caused by the self-weight of the tree, adding a tensile stress on the upper side and a balanced compressive stress on the lower side of the tilted stem. To account for this effect, four measurements were taken on each tree: one on the upper side and the others at 90° intervals around the trunk. The average BRLS per tree was then computed as the arithmetic mean of the four measurements. Averaging the four measurements eliminates the effect of bending due to the self-weight of the tree. All the measurements performed in this study are listed in Table 2. As a minimum bark thickness (~2 mm) is required to make accurate measurement with the extensometer sensor, the BRLS was only measured on trees with a sufficient bark thickness (from 11 to 15 trees per species, Table 3). Therefore, the minimum tree size sampled for BRLS depends on the species, so that the thicker the bark, the lower the minimum tree size sample for BRLS within a species.

**Table 2.** List of the variables measured in the study of six tropical tree species in the tropical rainforest of French Guiana

Measured traits	Unit	Method	*N*	*N*_tree_
Bark residual longitudinal strain (BRLS)	µstrain (µm m^–1^)	Extensometric sensor^*a*^	296	74
Bark basic density (BBD)	g cm^–3^	Water displacement method	500	125
Inner bark thickness	cm	Image analysis	500	125
Stem radius (stem diameter/2)	cm	Image analysis	500	125
Inner bark thickness:stem radius	Unitless ratio		500	125
Inner bark thickness:wood thickness	Unitless ratio		500	125

The units, the methodology, the number of measurements (four measurements per tree) (*N*), and the number of trees sampled (*N*_tree_) are given.

^*a*^ See Fournier *et al.* (1994) and Lehnebach *et al.* (2019).

**Table 3.** Characteristics of the samples and description of bark traits measured in the study of six tropical tree species in the tropical rainforest of French Guiana

	*N*	*N*_BRLS_	Stem diameter range (cm)	Bark basic density (BBD) range (g cm^−3^)	Bark thickness range (cm)	Bark residual longitudinal stress (BRLS) range (µstrain)
*Goupia glabra*	25	13	0.9–40.5	0.293–0.842 a	0.04–0.78 a	503–4046 a
*Jacaranda copaia*	20	12	1.6–40	0.222–0.644 b	0.12–0.82 a	249–4136 a
*Simarouba amara*	20	11	1–32.3	0.178–0.617 b	0.09–0.68 a	–765–1109 b
*Virola surinamensis*	20	12	1–38.9	0.177–0.417 cd	0.08–0.95 a	–1148 to –124 bc
*Cecropia obtusa*	20	11	1–26.5	0.168–0.323 d	0.07–0.77 a	–2244 to –5 c
*Pachira aquatica*	20	15	0.7–30.6	0.2–0.429 bc	0.08–1.27 a	–3702 to –1223 d

For each species, the observed range (minimum–maximum) of each trait is given.

Different letters indicate statistical differences between species. *N*, number of trees used to measure morphometry and density; *N*_BRLS_, number of trees used to measure residual bark strain.

### Collection of samples

Following the BRLS measurements, one bark sample (1×2–5 cm) was collected below each measurement location (four samples per tree), labeled, and sealed in plastic bags for further processing. Care was taken to collect samples containing outer bark. In the case of the smallest trees for which BRLS was not recorded, the side opposite to the direction of leaning was selected (position 1) and labeled with a vertical line drawn with a marker pen. Then, two transversal stem discs ~1 cm thick were cut. The upper side of both discs was labeled and the discs were sealed in plastic bags.

### Measurement of inner bark basic density

In the laboratory, each sample taken from the largest trees was longitudinally sectioned into two subsamples. One subsample was used to measure thickness and the other to measure inner bark basic density. For the smallest trees, one disc was selected and four samples of inner bark were extracted from the disc to measure basic density. Density was measured within 24 h after sampling in the field. After removing the outer bark, the green volume of the inner bark sample was estimated using the water displacement method with a high precision scale (Sartorius CP224S). After stabilization of the mass at 103 °C for 48 h, the dry weight of the sample was measured using the same scale. The inner bark basic density (BBD, g cm^−3^) was computed as the ratio of dry mass to green volume. For each tree, the BBD was computed as the arithmetic mean of four density measurements.

### Measurement of inner bark thickness and wood and pith radius

The transverse section of the bark samples was polished using a stationary polishing machine. The whole transverse section of the discs collected from the small trees was polished. The samples and stem discs were then digitized using a flatbed scanner (Canon LIDE 700F) at 1200 dpi. Both total bark thickness and inner bark thickness were measured directly on the bark samples from the largest trees. We measured pith radius, wood thickness, inner and outer bark thickness, and stem radius at four positions on the stem discs from the smallest trees. The first radius corresponded to position 1, and measurements at position 2, 3, and 4 were made 90° from the previous position moving clockwise. Measurements were carried out with Image J software (Rasband, 2012). An average pith radius (in centimeters) was computed for each species and applied to the largest tree. The wood thickness of the largest trees (in centimeters) was computed as the difference between the stem radius and the sum of pith radius and total bark thickness. For each tree, the individual inner bark and wood thickness was computed as the arithmetic mean of the four measurements.

### Statistical analysis

In order to assess how the geometric contribution of inner bark changes with tree size, we evaluated the allometric relationship between inner bark thickness and stem radius (i.e. stem diameter/2). Both variables were log-transformed and a standard major axis regression including species effect was fitted. A likelihood ratio test was used to detect different allometric exponents (i.e. differences in the slope of log-transformed variables) followed by pairwise comparison of species. The likelihood ratio (LR) was also used to test whether the relationship between bark thickness and stem radius was allometric (i.e. the slope differed from 1). We also assessed how cambial functioning (i.e. the rate of cells produced by the cambium on the phloem side versus the xylem side) changed with tree size by evaluating the allometric relationship between inner bark thickness and wood thickness and using the same statistical procedure. The statistical tests related to the allometric analysis were performed with the ‘smatr’ package (Warton *et al.*, 2012) implemented in R statistical software (R Development Core Team, 2016).

To assess the relative investment in inner bark across species with different relative bark thickness (i.e. inner bark thickness divided by stem radius) and in the inner bark to wood thickness ratio were assessed in the smallest (stem diameter <2.5 cm) and the largest (stem diameter >20 cm) diameter classes. Variations in bark properties with size were assessed by bivariate analysis of BRLS and BBD versus stem radius. Both BRLS and BBD were fitted to log10-transformed stem radius through ordinary least squares linear regressions. The use of *P*-values in this study follows the statement of the American Statistical Academy (ASA) (Wasserstein and Lazar, 2016), so that the wording relative to statistical significance is avoided. In that sense, the *P*=0.05 threshold differentiates the detection from the non-detection of statistical differences. Following the ASA statement, the estimated coefficients and associated *P*-values are also supplemented by confidence intervals.

## Results

### Bark size allometry

**Fig. 2.**
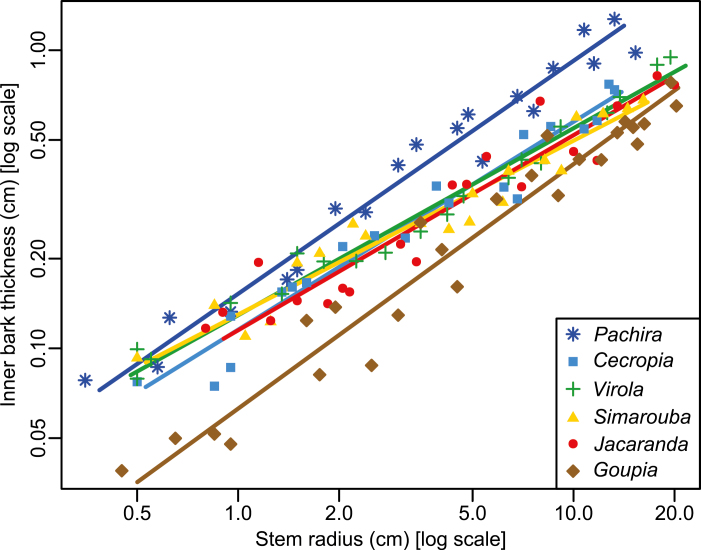
Allometric relationship between inner bark thickness and stem radius in six tropical tree species in the tropical rainforest of French Guiana. Each symbol represents a species. The regression lines result from the standard major axis regression models described in Table 2. (This figure is available in color at *JXB* online.)

The relationships between inner bark thickness and stem radius were log–log linear in all the species (Fig. 2). The overall slope (i.e. the allometric exponent) of this relationship was 0.66 and <1 (*r*= –0.73, df=123, *P*<0.001; Table 4), meaning that the relationship is allometric. The geometric contribution of bark thus decreased with increasing stem size. However, the allometric exponent differed between species (LR=32.82, df=5, *P*<0.001), ranging from 0.58 in *Simarouba* to 0.78 in *Pachira* (Table 4). We did not detect any differences between the allometric exponents for *Pachira* (0.78) and *Goupia* (0.82) (*P*>0.05), but the exponents did differ (*P*<0.01) from those estimated for *Virola* and *Simarouba* (0.63 and 0.58, respectively). The allometric exponents for *Jacaranda* and *Cecropia* were intermediate (0.65 and 0.7, respectively) and did not differ from the others (*P*>0.05).

**Table 4.** Overall and species-level parameters of the allometric relationships (standard major axis) between inner bark thickness and stem radius in six tropical tree species in the tropical rainforest of French Guiana.

Species	Intercept (CI)	Slope (CI)	*R* ^2^	*P*
Overall	–0.93 (–0.97; –0.9)	0.66 (0.63; 0.72)	0.85	**<0.001**
*Pachira aquatica*	–0.82 (–0.87; –0.77)	0.78 a (0.72; 0.85)	0.97	**<0.001**
*Cecropia obtusa*	–0.94 (–0.99; –0.89)	0.70 ab (0.63; 0.77)	0.95	**<0.001**
*Virola surinamensis*	–0.89 (–0.92; –0.85)	0.63 b (0.58; 0.68)	0.98	**<0.001**
*Simarouba amara*	–0.89 (–0.94; –0.83)	0.58 b (0.52; 0.65)	0.94	**<0.001**
*Jacaranda copaia*	–0.94(–1.00; –0.87)	0.65 ab (0.57; 0.74)	0.90	**<0.001**
*Goupia glabra*	–1.2 (–1.27; –1.13)	0.82 a (0.74; 0.90)	0.95	**<0.001**
		Species effect **<0.001**		

*P*-values (*P*) lower than 0.05 are in bold.

Different letters indicate statistical differences in slope among species. CI: confidence interval; R^2^, goodness of fit.

**Fig. 3.**
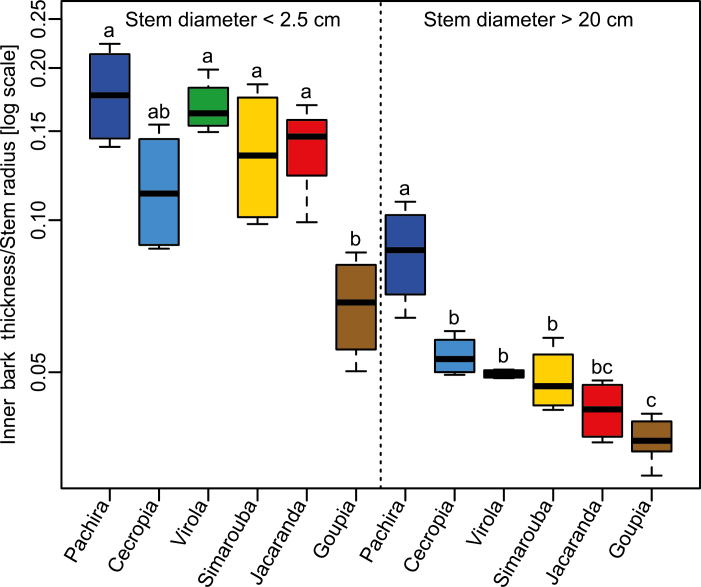
Across-species variation in relative bark thickness in large and small trees belonging to six tropical tree species in the tropical rainforest of French Guiana. Different letters indicate statistical differences between species. (This figure is available in color at *JXB* online.)

The ranking of species did not change with size: species with the thinnest or thickest bark when their stems were small also had the thinnest or thickest bark when their stems increased in size. This was highlighted by the variations in relative bark thickness across species and between small and large stems (Fig. 3). In small stems, the relative bark thickness in *Goupia* was lower than in the other species (*P*<0.01). Among these other species, no differences were observed, but the highest relative bark thickness was observed in *Pachira*. In the large stems, the relative bark thickness of *Pachira* was higher than in the other species (*P*<0.001). Among the other species, differences in relative bark thickness in large stems were more tenuous, although once more *Goupia* had the lowest relative bark thickness.

As mentioned in the Materials and methods, the relationship between bark thickness and stem radius informed us about changes in the geometric contribution of bark with tree size. The relationship between bark thickness and wood thickness can be computed by subtracting the radius of the pith. This relationship informs us about how the cambial functioning (namely the rate of cells produced by the cambium on the phloem side versus the xylem side) changes with tree size. The allometric analysis of bark thickness versus wood thickness revealed similar patterns to those obtained for the bark thickness/stem radius relationship (Supplementary Fig. S1 at *JXB* online), except that the allometric exponents were lower (Supplementary Table S1). The only exception was *Cecropia*, in which the ratio of bark thickness to wood thickness was the highest in small stems (Supplementary Fig. S2) and the allometric exponent was the lowest (Supplementary Table S1), due to its thick pith.

### Size-dependent variations in bark basic density

**Fig. 4.**
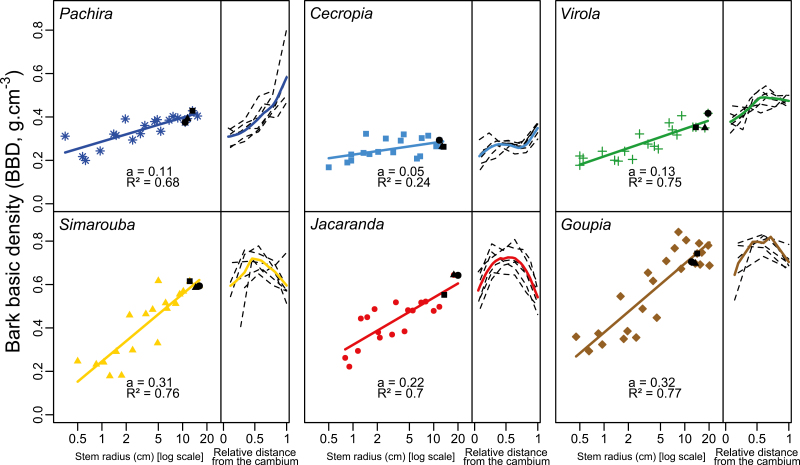
Ontogenetic and radial variation in bark basic density (BBD) in six tropical tree species in the tropical rainforest of French Guiana. For each species, BBD is plotted against the log-scaled stem radius; the slope value (a) and the goodness of fit (*R*^2^) are presented (left panels). For each species, the regression slope is statistically different from 0 (*P*<0.05). BBD is also plotted as a function of the relative distance from the cambium in three trees (two series of measurements per tree) (right panels), indicated on the overall regression by the black symbols (left panels). (This figure is available in color at *JXB* online.)

BBD increased with increasing stem radius in all the species (*P*<0.05) (Fig. 4). The highest rates of increase in BBD with stem radius were observed in *Simarouba*, *Jacaranda*, and *Goupia* (slope >0.09) and were 3–4 times higher than in *Pachira*, *Cecropia*, and *Virola* (slope <0.05) (Fig. 4). As bark thickness also increased with stem radius, BBD increased with bark thickness (data not shown). The BBDs measured here are a tissue-level average value, but it is important to note that bark density varies from the inner to the outer layers, as revealed by the bark density radial profiles (Fig. 4, right panels). Whereas BBD increased continuously from the cambium outward in *Pachira*, *Cecropia*, and *Virola*, the observed pattern was curvilinear in *Simarouba*, *Jacaranda*, and *Goupia*, with the highest BBD values recorded in the middle of the bark.

### Size-dependent variations in bark residual longitudinal strain

**Fig. 5.**
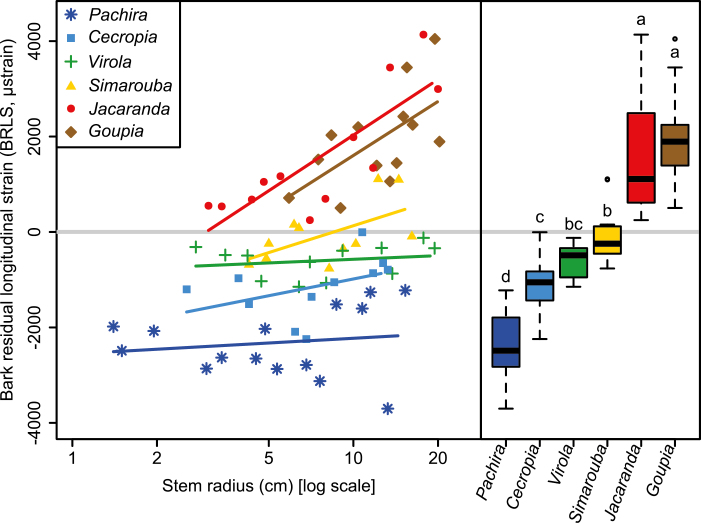
Ontogenetic variation in bark residual longitudinal strain (BRLS) in six tropical tree species in the tropical rainforest of French Guiana. BRLS is plotted against the log-scaled stem radius (SR) (left panel). Between-species differences in BRLS are also given (right panel). Different letters indicate statistical differences between species according to a Kruskal–Wallis test (χ ^2^=63.098, df=5, *P*<0.001). (This figure is available in color at *JXB* online.)

We observed three different patterns of variation in BRLS with increasing stem radius (Fig. 5). In *Goupia* and *Jacaranda*, BRLS was positive and increased strongly with increasing stem radius (*P*<0.05). In *Simarouba*, although the lower bound of the confidence interval of the slope was slightly negative [i.e. (–99;3839) Supplementary Table S2], BRLS tended to increase with stem radius from negative to positive values. Finally, in *Pachira*, *Cecropia*, and *Virola*, BRLS values were negative and no variations with stem radius were detected (*P*>0.05).

**Fig. 6.**
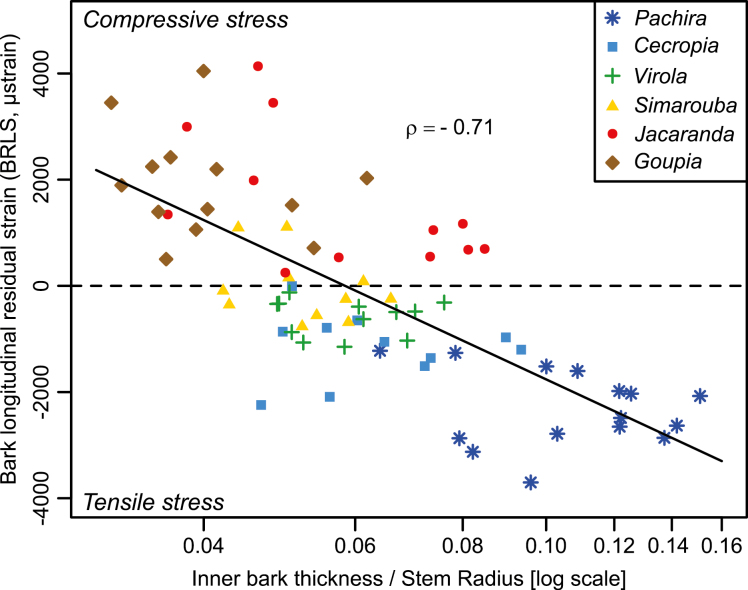
Variations in bark residual longitudinal strain (BRLS) as a function of relative bark thickness in six tropical tree species in the tropical rainforest of French Guiana. The dashed horizontal line separates compressive (*y*>0) and tensile (*y*<0) BRLS. Different symbols indicate different species. Spearman’s correlation coefficient (ρ=0.71, *P*<0.001) is shown. (This figure is available in color at *JXB* online.)

The relationship between BRLS and relative bark thickness was negative (ρ= –0.71, *P*<0.001) and showed that species with thin bark generally have compressive BRLS, while species with thicker bark produce tensile stress in bark (Fig. 6).

## Discussion

### Scaling of inner bark thickness with stem size

Studying a much broader species diversity, Rosell (2016) reported a similar distribution of bark thickness, indicating that our sampling is representative of other tropical rainforests. We observed a 2- to 2.5-fold difference in bark thickness between species with the thinnest and the thickest bark in both small and large stems. Our analysis shows that the ranking of species according to bark thickness for a given tree size is usually maintained throughout the life of the tree. This was clear in *Pachira* and *Goupia*, which had, respectively, the thickest and thinnest bark, and the highest and lowest relative bark thickness in both small and large stems. The ontogenetic variation in bark thickness was higher than variations between species. Overall, a 6-fold increase in bark thickness was observed with increasing stem size, whose magnitude clearly depended on the species considered, from 5-fold in *Simarouba* to 9-fold in *Goupia*.

Relationships between inner bark thickness and stem radius were all allometric both between and within species. When all six species were pooled, the mean allometric exponent was <1 (0.66) and very close to the value (0.70) reported by Rosell (2016). Within species, the allometric exponents were also <1 and ranged from 0.63 to 0.82. Interestingly, when studying tree species in dry and wet tropical forests, Poorter *et al.* (2014) reported allometric exponents ranging from 0.38 to 1.20 (70% were <1) based on measurements made on the whole bark (i.e. without accounting for the difference between inner and outer bark). This may mean that the allometry between outer bark thickness and stem radius differs from that between inner bark thickness and stem radius.

In our sample, we also found an allometric relationship between inner bark thickness and wood thickness, with an allometric exponent ranging from 0.5 to 0.77 (average 0.57). This indicates that at the cambium level, the ratio of phloem to xylem cambial production decreases with tree development whatever the species considered, with a lower rate of decrease in *Pachira* and *Goupia*.

While the two allometric relationships provided relatively similar results, they need to be interpreted in different ways. When we linked the scaling of bark thickness to the size of the whole stem, the allometry between bark and stem radius revealed decreasing involvement of bark in stem thickening with increasing stem size. On the other hand, the bark–wood allometry described the balance in cambial production between the inner bark and wood. The discrepancy observed between the two allometric relationships in *Cecropia* highlights the importance of the pith thickness in such an analysis. While the allometric exponent was relatively high (i.e. 0.70) when stem size was included, *Cecropia* had the lowest exponent relative to wood thickness (i.e. 0.5) and therefore its cambial production imbalance was increasingly in favor of wood as the stem increased in size.

### Size-dependent variation in inner bark density

In a study on branches of 90 species, Rosell *et al.* (2014) reported inner BBDs ranging from 0.17 g cm^−3^ to 0.86 g cm^−3^. The samples in our study covered the same range (0.168–0.842) even though only six species were sampled. Such a wide range of BBD values was obtained by taking ontogenetic variations into account. In all six species we sampled, BBDs systematically increased with increasing stem size. As bark thickness also increases with stem size, obviously BDD increases with bark thickness. Therefore, the negative correlation between BBD and bark thickness commonly observed at the interspecific level (Poorter *et al.*, 2014; Rosell *et al.*, 2014) does not hold among conspecific individuals of varying size. The magnitude of variation in BBD with stem size also depends on the species. In the present study, the greatest rates of increase were observed in *Goupia*, *Simarouba*, and *Jacaranda*, and were two to six times larger than those observed in *Pachira*, *Cecropia*, and *Virola*. The low ontogenetic variations in BBD were also associated with the lowest BBD in small stems, except in *Simarouba*, in which the BBD was relatively low in small stems but increased sharply with an increase in stem size. Radial profiles of inner bark density (Fig. 4) measured in the largest trees revealed substantial variation within the bark. *Pachira*, *Cecropia*, and *Virola* showed a monotonic increase in BBD outward, while *Simarouba*, *Jacaranda*, and *Goupia* showed a bell-shaped pattern, with the highest BBD values in the middle of the bark. Both patterns can be inferred from the spatial pattern of stone cell differentiation and parenchyma sclerification (Fig. 1; Lehnebach *et al.*, 2019). Sclerification occurs in the outer part of the inner bark and in strongly dilated regions in *Pachira*, *Cecropia*, and *Virola* (Clair *et al.*, 2019), causing the outward increase in BBD. In contrast, species in the second group are known to develop a substantial quantity of dense sclereid cells close to the cambium (Lehnebach *et al.*, 2019). The early differentiation of sclereids and the dilatation in the outer part of the inner bark explain the curvilinear BBD pattern observed in *Simarouba*, *Jacaranda*, and *Goupia*.

### Variations in residual strain and relationship to bark basic density and allometry

As expected, species with fibers organized like a trellis (*Pachira*, *Cecropia*, *Virola*, and *Simarouba*) presented negative inner BRLS corresponding to tensile stress, whereas species with no trellis (*Jacaranda* and *Goupia*) presented positive BRLS (Clair *et al.*, 2019; Lehnebach *et al.*, 2019) corresponding to compressive stress. The highest allometric exponents (inner bark/wood thickness) were observed in *Goupia* and *Pachira*, two species with contrasting average BRLS. The value of the allometric exponent is not related to the average BRLS of the species. However, we found a relationship between relative bark thickness and average BRLS (Fig. 6). Indeed, the highest ratio between bark thickness and stem radius was observed in *Pachira*. It is noteworthy that, after accounting for the effect of pith (i.e. by calculating the ratio of bark thickness to wood thickness), the relative bark thickness of *Cecropia* was the highest and was associated with tensile stress, as in *Pachira*. This suggests that the two species achieved more negative BRLS while maintaining a relatively high proportion of bark in relation to wood. This is in agreement with the less negative BRLS observed in *Virola*, whose bark fibers are also organized like a trellis but whose bark is relatively thinner than that observed in *Pachira* and *Cecropia*.

While BRLS does not vary with size within species with tensile stress (except in *Simarouba* whose BRLS showed a slight increasing trend from tensile to compressive stress), we observed a marked increase in BRLS with ontogeny in *Goupia* and *Jacaranda.* This strong ontogenetic increase in BRLS was associated with a marked ontogenetic increase in BBD. We previously proposed that compressive stress in bark results from the sclerification of parenchyma cells (Lehnebach, 2019). Although we did not provide any mechanical evidence for such a phenomenon, the association between increasing BRLS and BBD that we found in the present study strongly reinforces this hypothesis. The shift from tensile to compressive stress observed in *Simarouba* is consistent with the substantial increase in BBD we also observed in this species and points to a possible ontogenetic shift from tensile to compressive stress in bark.

The observed increase in BBD also revealed differences between the rate of sclerification on one hand and the rate of dilatation growth on the other hand. Indeed, to face the increase in the stem perimeter resulting from wood growth, bark has to extend tangentially. Whether achieved by cell enlargement and/or by the division of thin-walled parenchyma cells, the tangential extension of bark should reduce the basic density of the dilating bark. On the other hand, cambial growth adds new fiber-rich layers of inner bark. These two processes should result in a more or less constant BBD with size, which is contrary to our observations. The strong ontogenetic increase in BDD we observed in some species is likely to be due to sclerification occurring at a higher rate than that of dilatation.

While ontogenetic BBD variations are lower in species with constant tensile stress (i.e. *Pachira*, *Cecropia*, and *Virola*), they are still positive. This suggests that sclerification increases during growth and progressively adds compressive stress. Yet, in these species, we observed constant tensile stress rather than an increase in BRLS toward compressive values. This discrepancy suggests that the source of tension (namely the deformation of the trellis) increases during growth, counteracting the effect of sclerification.

### Implications for mechanisms of stem posture control

In previous works we showed that the mechanical stress generated in bark during growth can be the basis of a mechanism for the control of stem posture (Clair *et al.*, 2019). This was directly demonstrated on small tilted stems of *Pachira*, *Cecropia*, *Virola*, and *Simarouba* (Clair *et al.*, 2019). The mechanism involves a source of longitudinal tensile stress in the bark (interaction between wood radial growth and fibers organized like a trellis) and a source of asymmetry (eccentric growth). In tilted trees, eccentricity has been shown to be particularly strong in species that rely on bark for posture control (Ghislain *et al.*, 2019). In vertical trees, the source of asymmetry is no longer active, but the source of tensile stress still is. This was confirmed by our observation that bark stress is tensile in the earliest growth stages in the four above-mentioned species (Fig. 5). However, in *Simarouba*, this tension is reduced and progressively becomes compression due to sclerification. In *Pachira*, *Cecropia*, and *Virola*, tensile stress is maintained during tree growth, although at different levels.

The performance of the mechanism also depends on the thickness of the bark. Interestingly, species with higher relative bark thickness also displayed more tensile bark stress (Fig. 6). Our study also confirmed that the relative thickness of bark decreases with stem size (Fig. 2), suggesting that the performance of the mechanism is reduced in larger stems. However, the reduction was less marked in *Pachira*, the species with the highest relative bark thickness and the most tensile stress. Taken together, these observations suggest that the decrease in performance is mitigated in the species that relies most on this mechanism.

Species such as *Cecropia*, *Virola*, or *Simarouba* probably rely on bark for posture control in their early development stages, when the mechanical contribution of bark is dominant (Niklas, 1999; Rosell and Olson, 2014). However, the performance of the bark mechanism decreases steeply with increasing size. In later stages, the usual mechanism based on tension wood takes over, as demonstrated by measurements of residual strains (Clair *et al.*, 2019). The same measurements in *Pachira* did not show any strong residual strain in tension wood (Clair *et al.*, 2019) (Ghislain *et al.*, 2019), strongly suggesting that in this species, posture control is supported by the bark even in late development stages.

Theoretically, the performance of the mechanism does not depend on the sign of stress (tensile or compressive). For instance, mechanisms based on the action of wood are based on either tension wood or compression wood, with similar efficiency (Alméras *et al.*, 2005). *Jacaranda* and *Goupia* develop strong bark compressive stress, leading one to wonder if this could be the basis of a hypothetical mechanism for the control of stem posture. Our results do not support this hypothesis. First, such a mechanism would require a source of asymmetry, namely more sclereids in the lower part of the bark of a tilted stem. No evidence for such asymmetry has yet been put forward. Moreover, the largest compressive stresses were associated with the thinnest barks, both within and between species. In the early stages, when the relative thickness of bark is still high, compressive stress is low. In late stages, when the bark is sclerified and therefore in compression, its relative thickness is low. Thus, this hypothetical mechanism would not be performant. It is likely that the compressive stress in the bark of *Jacaranda* and *Goupia* has no specific biomechanical function and is simply the result of the growth of sclereids, whose primary function is defense against biological aggression (Wainhouse *et al.*, 1990; Thorpe and Day, 2002).

## Supplementary data

Supplementary data are available at *JXB* online.

Fig. S1. Allometric relationship between inner bark thickness and wood thickness in six tropical tree species in the tropical rainforest of French Guiana.

Fig. S2. Across-species variation in relative bark thickness (inner bark thickness divided by wood thickness) in large and small trees belonging to six tropical tree species in the tropical rainforest of French Guiana. 

Table S1. Overall and species-level parameters of the allometric relationships (standard major axis) between inner bark thickness and wood thickness.

Table S2. Parameters of the regression between bark residual longitudinal strain (BRLS) and the log of stem radius of six tropical tree species in the tropical rainforest of French Guiana.

## Supplementary Material

eraa070_suppl_supplementary_figures_S1_S2_tables_S1_S2
