# Supplementary material for: How does bark contribution to postural control change during tree ontogeny? A study of six Amazonian tree species
Source: J Exp Bot. 2020 Feb 13;71(9):2641–9. doi: 10.1093/jxb/eraa070 (PMC7210755; doi:10.1093/jxb/eraa070)
Supplement: eraa070_suppl_supplementary_figures_S1_S2_tables_S1_S2 [file eraa070_suppl_supplementary_figures_s1_s2_tables_s1_s2.pdf]

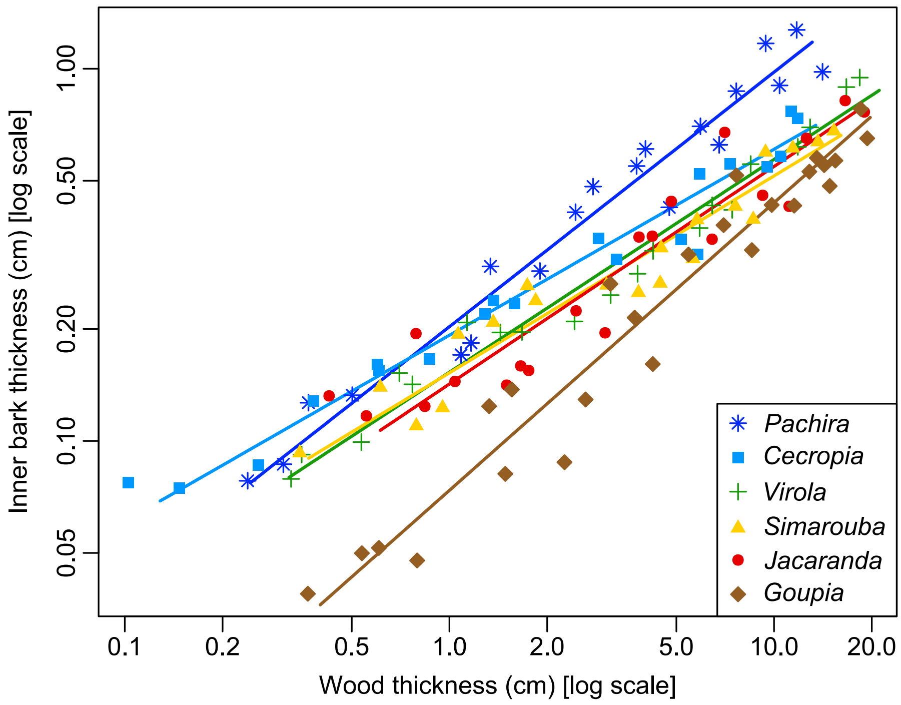

Figure S1: Allometric relationship between inner bark thickness and wood thickness for six tropical tree species from the tropical rainforest of French Guiana. Each color and symbol represents a species. The regression lines result from the standard major axis regression models described in Table S1.

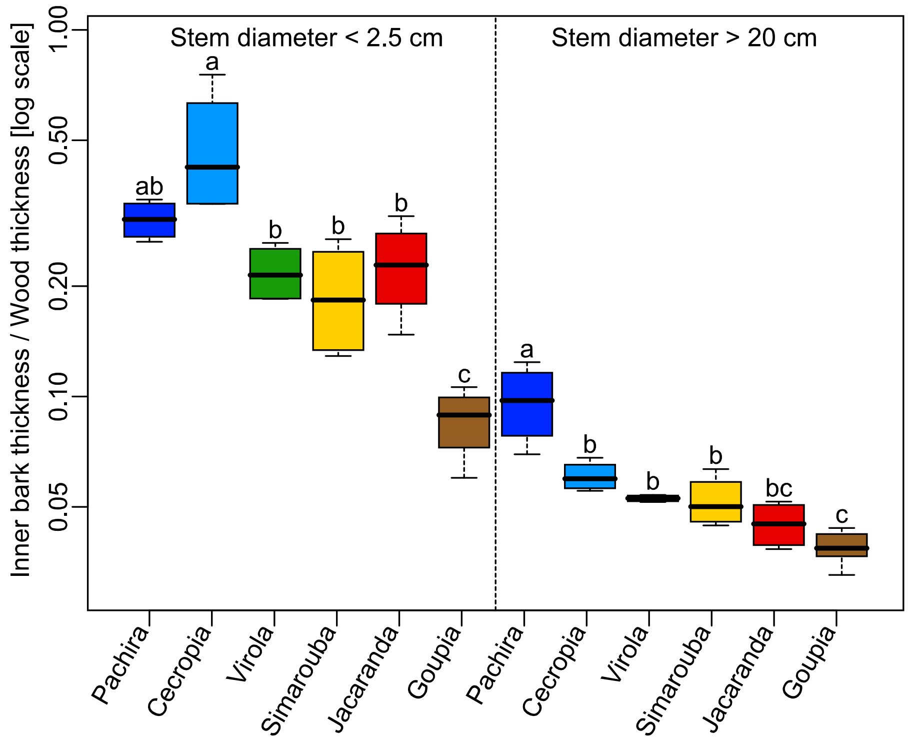

Figure S2: Across-species variation in relative bark thickness (inner bark thickness divided by wood thickness) in big and small trees for six tropical tree species from the tropical rainforest of French Guiana. Different letters highlight significant differences between species.

Table S1: Overall and species-level parameters of the allometric relationships (standard major axis) between inner bark thickness and wood thickness. P-values (*P*) lower than 0.05 are bolded. Different letters highlight statistical differences in slope among species.

Species	Intercept [CI]	Slope [CI]	R^2	*P*
Overall	-0.82 [-0.85; -0.78]	0.57 [0.53;0.61]	0.81	< **0.001**
Pachira aquatica	-0.69 [-0.74; -0.65]	0.68[ab] [0.63;0.74]	0.96	< **0.001**
Cecropia obtusa	-0.72 [-0.74; -0.69]	0.5[c] [0.46;0.54]	0.96	< **0.001**
Virola surinamensis	-0.82 [-0.85; -0.78]	0.57[c] [0.53;0.62]	0.97	< **0.001**
Simarouba amara	-0.82 [-0.868; -0.77]	0.53[c] [0.5;0.66]	0.93	< **0.001**
Jacaranda copaia	-0.85 [-0.917; -0.78]	0.58[bc] [0.5;0.68]	0.88	< **0.001**
Goupia glabra	-1.13 [-1.2; -1.1]	0.77[a] [0.71;0.85]	0.95	< **0.001**
		Species effect < **0.001**		

CI: confidence interval. *P*: p-value. R^2: goodness of fit.

Table S2: Parameters of the regression between bark residual longitudinal strain (BRLS) and log of stem radius for six tropical tree species from the tropical rainforest of French Guiana. P-values (*P*) lower than 0.05 are bolded.

Species	Intercept	Slope	R^2	*P*
Pachira aquatica	-2 556[-3558;-1554]	329[-946;1603]	0.02	0.59
Cecropia obtusa	-2 150[-3792;-508]	1 166[-700;3032]	0.09	0.19
Virola surinamensis	-826[-1608;-42]	253[-598;1104]	0.04	0.52
Simarouba amara	-1 738[-3559;82]	1 870[-99;3839]	0.26	0.06
Jacaranda copaia	-1 843[-3568;-117]	3 874[1996;5751]	0.65	< **0.001**
Goupia glabra	-2 155[-5666;1356]	3 759[550;6967]	0.32	**0.026**

P: p-value. R^2: goodness of fit.
